# Successful Production and Ligninolytic Activity of a Bacterial Laccase, Lac51, Made in *Nicotiana benthamiana via* Transient Expression

**DOI:** 10.3389/fpls.2022.912293

**Published:** 2022-05-13

**Authors:** André van Eerde, Anikó Várnai, Yanliang Wang, Lisa Paruch, John-Kristian Jameson, Fen Qiao, Hans Geir Eiken, Hang Su, Vincent G. H. Eijsink, Jihong Liu Clarke

**Affiliations:** ^1^NIBIO - Norwegian Institute of Bioeconomy Research, Ås, Norway; ^2^Faculty of Chemistry, Biotechnology and Food Science, Norwegian University of Life Sciences (NMBU), Ås, Norway; ^3^Institute of Plant Protection, Chinese Academy of Agricultural Sciences, Haidian, China

**Keywords:** bacterial laccase, lignin degrading enzymes, transient expression, tobacco, plant biotechnology

## Abstract

Giant panda could have bamboo as their exclusive diet for about 2 million years because of the contribution of numerous enzymes produced by their gut bacteria, for instance laccases. Laccases are blue multi-copper oxidases that catalyze the oxidation of a broad spectrum of phenolic and aromatic compounds with water as the only byproduct. As a “green enzyme,” laccases have potential in industrial applications, for example, when dealing with degradation of recalcitrant biopolymers, such as lignin. In the current study, a bacterial laccase, Lac51, originating from *Pseudomonas putida* and identified in the gut microbiome of the giant panda’s gut was transiently expressed in the non-food plant *Nicotiana benthamiana* and characterized. Our results show that recombinant Lac51 exhibits bacterial laccase properties, with optimal pH and temperature at 7–8 and 40°C, respectively, when using syringaldazine as substrate. Moreover, we demonstrate the functional capability of the plant expressed Lac51 to oxidize lignin using selected lignin monomers that serve as substrates of Lac51. In summary, our study demonstrates the potential of green and non-food plants as a viable enzyme production platform for bacterial laccases. This result enriches our understanding of plant-made enzymes, as, to our knowledge, Lac51 is the first functional recombinant laccase produced in plants.

## Introduction

Laccases (EC 1.10.3.2, benzenediol:oxygen oxidoreductases) are a group of blue multi-copper oxidoreductases which are able to catalyze the oxidation of phenols and aromatic biopolymers (e.g., lignin) using molecular oxygen. Laccases, e.g., Lac51, a bacterial laccase originating from the gut microbe *Pseudomonas putida*, are also proposed to play important roles in helping giant panda (*Ailuropoda melanoleuca*) to subsist on bamboo as their almost exclusive diet, by facilitating the oxidation of lignin moieties, thereby aiding the digestion of bamboo (Zhu et al., 2011; Fang et al., 2012).

Being versatile and eco-friendly enzymes (i.e., not producing toxic peroxide intermediates), laccases have received much attention from scientists and industry. They have been widely applied in food, pulp and paper processing, textile industries, and bioremediation (Chauhan et al., 2017; Yang et al., 2017). Besides, there is a growing interest in their potential utilization for treatment of recalcitrant biomass, such as lignocellulose materials.

In nature, laccases are widely distributed among plants, fungi, bacteria, and insects (Ausec et al., 2011). The most intensively studied ligninolytic laccases and the most applied industrial laccases are from fungi, especially the white-rot basidiomycetes (Martinez et al., 2017). Fungal laccases have a high redox potential and have been used in varying industrial applications, such as food processing, beverage stabilization, and pulp bleaching (Martinez et al., 2017). Unfortunately, fungal laccases are sensitive to temperature and alkaline conditions, which limits their use in industrial applications, where harsh conditions are normally applied in processes such as biomass pretreatment and pulp and paper bleaching (Martinez et al., 2017). In such processes, bacterial laccases could become an alternative as most of them exhibit better tolerance to a broad pH range and high thermostability. For example, the well-known CotA laccase, a coat protein of *Bacillus subtilis*, displays high thermostability with a half-life of inactivation at 80°C of about 4 h (Martins et al., 2002). Furthermore, a laccase isolated from γ-proteobacterium JB works optimally at 55°C and is highly alkali-tolerant, being stable in the pH range 4–10 (Singh et al., 2007).

Owing to their advantageous physiochemical properties, bacterial laccases may become attractive biocatalysts for diverse industrial applications, including lignin processing. Lignin degradation is an important step to facilitate the ability of downstream oxidases to act on the lignin, thus creating access to plant carbohydrates. To achieve lignin degradation, low-molecular-weight small molecules (e.g., ABTS, HBT, and syringaldazine) could act as redox shuttles between the laccase and the lignin (Chen et al., 2012). By using low-molecular-weight redox mediators, such as 2, 2′-azino-bis (3-ethylbenzothazoline-6-sulfonate) (ABTS) and 1-hydroxybenzotriazole (HBT), the range of substrates a laccase can catalyze can be greatly expanded (Chauhan et al., 2017). Actinomycetes, particularly *Streptomyces* species which carry small two-domain laccases have been shown to have high lignin-degrading capacity when supplied with redox mediators (Majumdar et al., 2014). In addition, a *Comamonas* sp. B-9 isolated from eroded bamboo slips exhibits high lignin-degrading activity when grown on kraft lignin as carbon source, which has been attributed to secreted enzymes that include laccases (Chen et al., 2012).

Heterologous expression of bacterial laccases has mostly been done using *Escherichia coli* as the production host (Martins et al., 2002; Suzuki et al., 2003; Wu et al., 2010; Fang et al., 2012). This approach may suffer from low yields or formation of inclusion bodies, complicating downstream purification (Suzuki et al., 2003). Plants have been used for heterologous production of various industrial enzymes (Kwon et al., 2018) due to the low-cost and flexible production scalability. Indeed, plants have been exploited for expression of different laccases derived from plants and fungi (Hood et al., 2003; Wang et al., 2004; Sonoki et al., 2005; Ligaba-Osena et al., 2017; Preethi et al., 2020), but hardly for laccases of bacterial origin.

In this study, we aimed at transiently expressing a bacterial laccase, denoted as Lac51 and originating from a gut microbe of the giant panda (Fang et al., 2012), using the plant *Nicotiana benthamiana*, and to investigate the enzymatic properties of the plant-derived Lac51. Next to assessing substrate oxidation, an LC–MS–MS method was used to investigate the fate of substrates 2,6-dimethoxyphenol (2,6-DMP) and 3,4-dihydroxybenzaldehyde (3,4-DHBA) upon oxidation by plant-produced Lac51, thus generating insight into product formation by this laccase. The functional capability of the enzyme to modify lignin was assessed using selected lignin monomers.

## Materials and Methods

### Lac51 Gene Synthesis

The *lac51* gene (GenBank accession number JN867369.1) from *Pseudomonas putida* (strain L, GenBank accession number AY450556.1; Fang et al., 2012) was chemically synthesized without its native signal peptide by GeneArt (New York, United States) after codon optimization for expression in *Nicotiana benthamiana*. The original TAT signal peptide at the N-terminus was replaced with the plant-derived signal peptide of Barley α-amylase (GenBank accession number CAX51374) linked with a His_6_-tag on the N-terminal end of *lac51*.

### Construction of the Lac51 Plant Expression Vector

**Figure 1:**
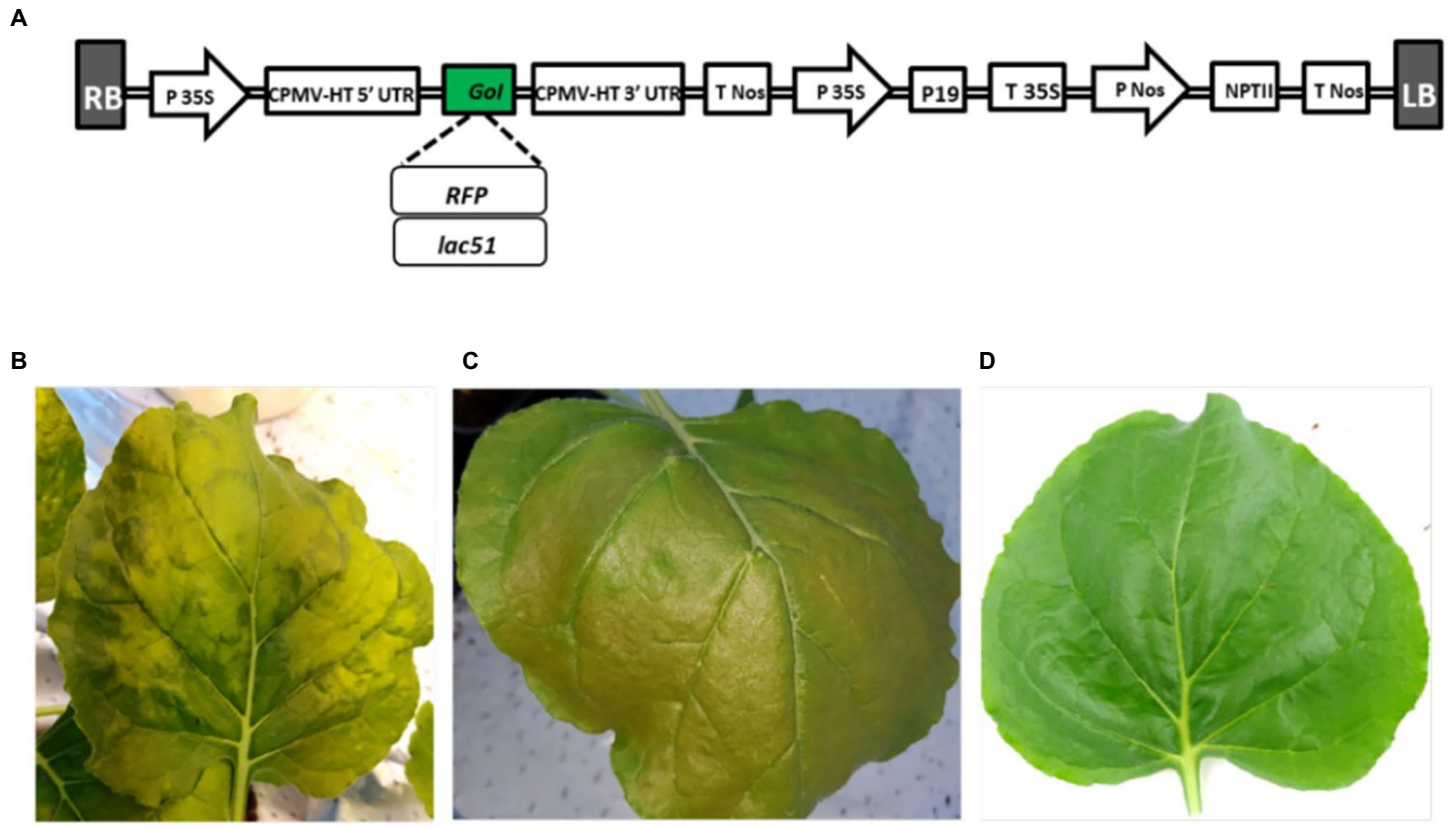
Expression of Lac51. **(A)** Schematic illustration of *lac51* and red fluorescent protein (*RFP*) gene expression vectors. The Lac51-encoding gene was introduced into the pEAQ-HT-DEST1 vector using Gateway cloning technology. RB and LB, right and left borders; CPMV-HT 5′ UTR and 3′ UTR, 5′ and 3′ untranslated regions derived from Cowpea mosaic virus (CPMV); P 35S and T 35S, 35S promoter and terminator; P Nos and T Nos, nopaline synthase promoter and terminator; P19, suppressor of gene silencing; NPTII, neomycin phosphotransferase II gene conferring kanamycin resistance. **(B–D)** Phenotype of *Nicotiana benthamiana* leaves expressing *lac51* **(B)** and the *rfp* reporter gene **(C)**, where the latter gives a distinguishable red color, at 5 dpi, as compared to a leaf infiltrated only with buffer as control **(D)**.

The *lac51* coding region was inserted into the plant transient expression vector pEAQ-*HT*-DEST1 (Sainsbury et al., 2009), as shown in Figure 1, using a Gateway cloning system provided by Life Technologies (BP&LR Clonase II kit, donor vector pDONR™/Zeo, Invitrogen, United States). The Gateway cloning BP and LR reactions (Karimi et al., 2002) were conducted based on the manufacturer’s protocol, as described previously (Dobrica et al., 2017). Like the *lac51* gene expression vector, a vector containing the red fluorescent protein (*rfp*) reporter gene was constructed.

### Agrobacterium-Mediated Transient Expression in *Nicotiana benthamiana*

The plant expression vectors pEAQ-*HT*-DEST1/RFP and pEAQ-*HT*-DEST1/Lac51 were introduced into ElectroMAX™ *Agrobacterium tumefaciens* LBA4404 cells (Invitrogen, United States) by electroporation using a BTX ECM 630 electroporator (BTX-Division of Harvard Apparatus, United States), with the parameters 2.0 kV, 200 Ω, and 25 μF, as described by Clarke et al. (2008). *Agrobacterium* LBA4404 harboring the expression vectors was cultured on LB medium supplemented with 50 mg L^−1^ kanamycin and incubated at 28°C for 72 h. The transformed clones were verified by colony PCR using the gene-specific-attB1/attB2 primer pair (attB1_lac51PSP 5′-GGGGACAAGTTTG TACAAAAAAGCAGGCTAT GGCTAACAAG CACCTGAG-3′; attB2_lac51PSP 5′-GGGGACC ACTTTGTACAAG AAAGCTGGGTCTACTTCA CCTCAATAGCAG-3′).

For transient expression of the *lac51* and *rfp* genes, *Agrobacterium* cells harboring pEAQ-*HT*-DEST1/RFP or pEAQ-*HT*-DEST1/Lac51 were cultured and prepared as described earlier (Clarke et al., 2017; Dobrica et al., 2017) and infused into *N. benthamiana*, using an in-house vacuum-based agroinfiltration protocol as described previously (Sainsbury et al., 2009). Around 4–6--week-old tobacco plants grown in the growth chamber under a photoperiod of 16 h light and 8 h dark with a temperature of 22°C were subjected to agroinfiltration, using a 2 ml syringe without needle on the underside of the leaves, basically as described by Dobrica et al. (2017). After the agroinfiltration, the *N. benthamiana* plants were grown further in a greenhouse maintaining the growth conditions and harvested after n—days (representing dpi n—m).

### Protein Extraction, Western Blot, and Purification

Protein extraction and Western blot analysis were done as before (Gottschamel and Lössl, 2016), with the following modifications. Briefly, for total protein analysis, frozen and grinded leaf samples were homogenized in pre-cold extraction buffer [0.7 M Sucrose, 0.5 M Tris–HCl pH 9.4, 50 mM EDTA, 0.1 M KCl, 2% β-mercaptoethanol (β-ME), 1x Complete protease inhibitor (CPI; Roche, Switzerland)]. After addition of an equal volume of phenol, mixing and incubation on a shaker for 10 min, and centrifugation at 5,500 *g* for 10 min at 4°C, the upper green phase was recovered and re-extracted with cold extraction buffer, mixed and incubated on a shaker for 10 min, and centrifuged at 5,500 *g* for 10 min at 4°C. The upper green phase was collected, and proteins were precipitated by overnight incubation with four volumes of 0.1 M NH_4_-acetate in methanol at −20°C. The pellet was recovered after centrifugation at 10,000 *g* for 10 min at 4°C, washed with 0.1 M NH_4_-acetate in methanol, air-dried, and dissolved in 1% SDS. The total soluble proteins were obtained by incubating the grinded leaf materials. After incubation for 15 min on ice, the supernatant was retained after centrifugation at 20,000 *g* for 30 min at 4°C. The protein concentration was determined with the Bradford assay (BioRad, United States) using bovine serum albumin (BSA) as standard.

For Western blot analysis, denatured protein samples were separated by electrophoresis in 4–12% SDS-polyacrylamide gels (Invitrogen, United States) and transferred to nitrocellulose membranes (Invitrogen, United States). The membranes were incubated with TBS-T solution (20 mM Tris–HCl pH 7.6, 137 mM NaCl, 0.1% Tween-20) containing 5% BSA as blocking buffer and subsequently treated with the primary and secondary antibodies diluted in TBS-T solution with 1% BSA. The recombinant proteins were detected with 1:5,000 diluted polyclonal anti-polyHistidine antibody produced in mouse, 1:20,000 diluted anti-AP-conjugated mouse IgG secondary antibody (Promega, United States) and a colorimetric reaction using the AP color development kit (Bio-Rad, United States). Protein samples prepared from tobacco leaves infiltrated with buffer were used as negative control.

For purification, powdered Lac51-containing leaf material was mixed with extraction buffer (0.15 M sodium phosphate buffer pH 8.0, 0.3 M KCl, 20 mM β-ME), filtered through Miracloth (Merck, Darmstadt, Germany) and centrifuged at 25,000 *g*, Lac51 was captured from the resulting supernatant by affinity chromatography using Ni^2+^-NTA beads (Qiagen, Hilden, Germany), washed with wash buffer (0.1 M sodium phosphate buffer pH 8.0, 0.3 M KCl, 10 mM imidazole), and eluted with elution buffer (0.1 M sodium phosphate buffer pH 8.0, 0.3 M KCl, 0.3 M imidazole). The eluted protein was concentrated using a 30 kDa MWCO Macrosep ultrafiltration device (Pall, Port Washington, United States) and buffer was exchanged to 0.05 M sodium phosphate buffer pH 8.0, 0.1 M KCl.

### Enzyme Activity Determination Assays

Laccase activity was assessed with 2,2′-azino-bis (3-ethylbenzothiazoline-6-sulphonic acid; ABTS, Roche diagnostic, Mannheim, Germany) and syringaldazine (Thermo Scientific, Rockford, United States) as model substrates. As a reference, *Tv*Lac from *Trametes versicolor* (Sigma Aldrich, Saint Louis, MO, United States) was used. Enzymatic reactions were set up in 96-well plates with 200 μl total reaction volumes containing 0.1 mM substrate (ABTS or syringaldazine) in 50 mM Na-acetate buffer pH 5.0. The plate was incubated in an Eppendorf Thermomixer C (Eppendorf AG, Hamburg, Germany) at 37°C and 400 rpm and scanned with a Synergy H4 Hybrid plate reader (BioTek, Winooski, VT, United States) at 0.5, 5, 10, 20, and 30 min. Reactions were scanned at 350 and 740 nm for detecting ABTS and the ABTS radical, respectively, and at 530 nm for detecting the oxidized form of syringaldazine. In addition, the full spectrum of the final reactions, from 300 nm to 800 nm, was recorded after 31 min (see Supplementary Figure S1).

### Determination of pH and Temperature Optima of the Plant Produced Lac51

To evaluate the biochemical activity of Lac51 in different conditions, syringaldazine was used as substrate. The assay system contained Cu^2+^-saturated Lac51, 50 mM Na-acetate pH 5.0, and 30 μM syringaldazine in a final volume of 0.1 ml. The absorbance was measured at 530 nm for over 30 min at 37°C. The effect of pH on laccase activity was determined using the following buffers: 50 mM Na-acetate buffer (pH 3.0–5.0), 50 mM K_2_HPO_4_-KH_2_PO_4_ buffer (pH 6.0–8.0), and 50 mM Tris–HCl buffer (pH 7.0–9.0). The effect of temperature was measured from 25 to 50°C in 50 mM Tris–HCl (pH 7.0).

### Enzymatic Reaction With Lignin Monomers

Seven phenolic compounds were tested as potential substrates of Lac51: 2,6-DMP, 3,4-dimethoxybenzalcohol (3,4-DMBA), 3,4-dihydroxybenzaldehyde (3,4-DHBA), 4-methoxybenzylalcohol (4-MBA), gallic acid (GA), vanillin (V), and guaiacol (G), all purchased from Sigma Aldrich (Saint Louis, MO, United States). Enzymatic reactions were carried out in 2-ml Eppendorf tubes with 0.2 ml of total volume. The reactions contained 3 mM substrate and Lac51 or *Tv*Lac in 10 mM BisTris-HCl buffer pH 6.5. The reactions were incubated in an Eppendorf Thermomixer C (Eppendorf AG) at 37°C and 800 rpm for 24 h. After 24 h, the reactions were filtered through a 96-well filter plate equipped with 0.45-μm filter membrane (Merck Millipore Ltd., Tullagreen Carrigtwohill, Ireland) and analyzed with high performance liquid chromatography (HPLC) using a Dionex Ultimate 3000 (Dionex, Sunnyvale, United States) equipped with an Agilent Eclipse Plus C18 RRND 1.8 μm, 2.1 × 50 mm column equilibrated at 30°C and coupled to a Dionex Ultimate 3000 RS VWD UV detector and an LTQ Velos Pro Mass Spectrometer detector (Thermo Scientific, Rockford, United States). The equipment used was as follows: Column: Agilent Eclipse Plus C18 RRND 1.8 μm, 2.1 × 50 mm column; column temperature: 30°C; flow rate: 0.4 ml/min; detection: UV coupled to MS with direct flow; UV–Vis recorded at 315, 450, and 600 nm; MS scan in the *m/z* range 85–1,200. Initially, the column was conditioned with a mixture of 5% eluent B (100% ACN with 0.1% TFA) and 95% eluent A (100% H_2_O with 0.1% TFA) for 0.5 min, followed by a linear gradient increasing eluent B from 5 to 95% over 9.5 min. The concentration of eluent B was kept at 95% for 5 min, then increased linearly to 100% over 1 min. Next, the concentration of eluent B was set back to 5% with a linear gradient over 0.5 min. The chromatographic profile ended with reconditioning the column at 5% eluent B for 9 min before the next injection.

## Results

### Expression and Purification of Lac51 in *Nicotiana benthamiana*

**Figure 2:**
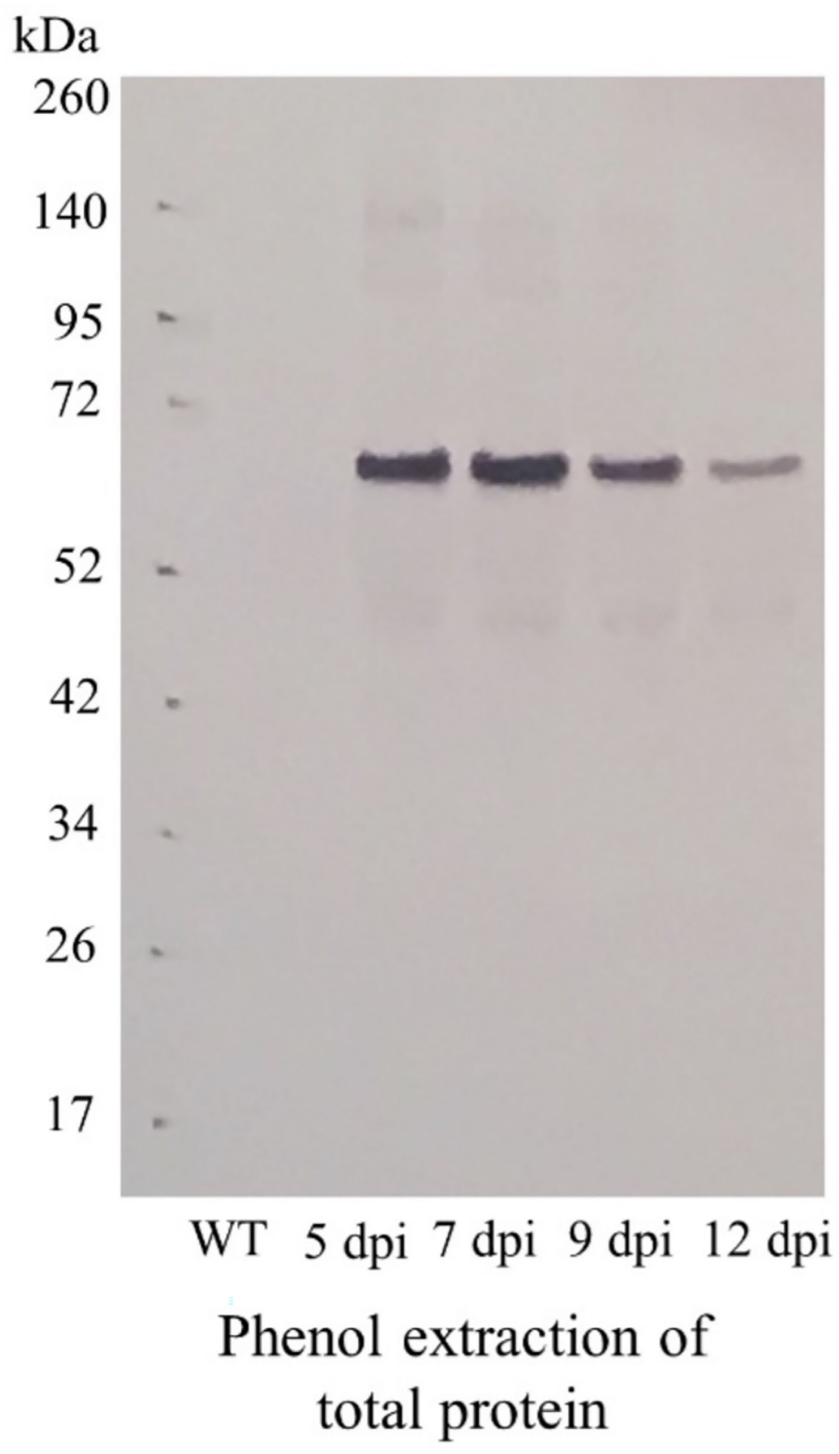
Accumulation of Lac51 protein in *Nicotiana benthamiana* leaves. Immunoblot analysis of total protein extracts from *N. benthamiana* leaves infiltrated with the *lac51* construct, harvested 5, 7, 9, and 12 days after infiltration (dpi) or non-infiltrated leaves (WT).

The *lac51-* and *rfp*-expressing vectors (Figure 1A) were constructed and successfully introduced in *N. benthamiana* plants (leaves) using vacuum infiltration. Five days after infiltration (dpi), phenotypical alterations (Figures 1B,C) could be found when compared with the wild-type control (infiltrated with buffer; Figure 1D). To identify the optimal harvest time, material from *lac51*-expressing plant leaves was harvested at different days post infiltration (5, 7, 9, and 12) for analysis of Lac51 accumulation. Total proteins were isolated and subjected to Western blot analysis (Figure 2), which showed accumulation of Lac51, peaking at 7 dpi (Figure 2A). After 7 dpi, the amount of target protein declined rapidly, potentially indicating protein instability. Then, Lac51 was extracted and purified from leaves harvested at 7 dpi, using Ni-affinity chromatography and subsequently used for activity assays.

### Lac51 Enzyme Activity on Model Substrates

**Figure 3:**
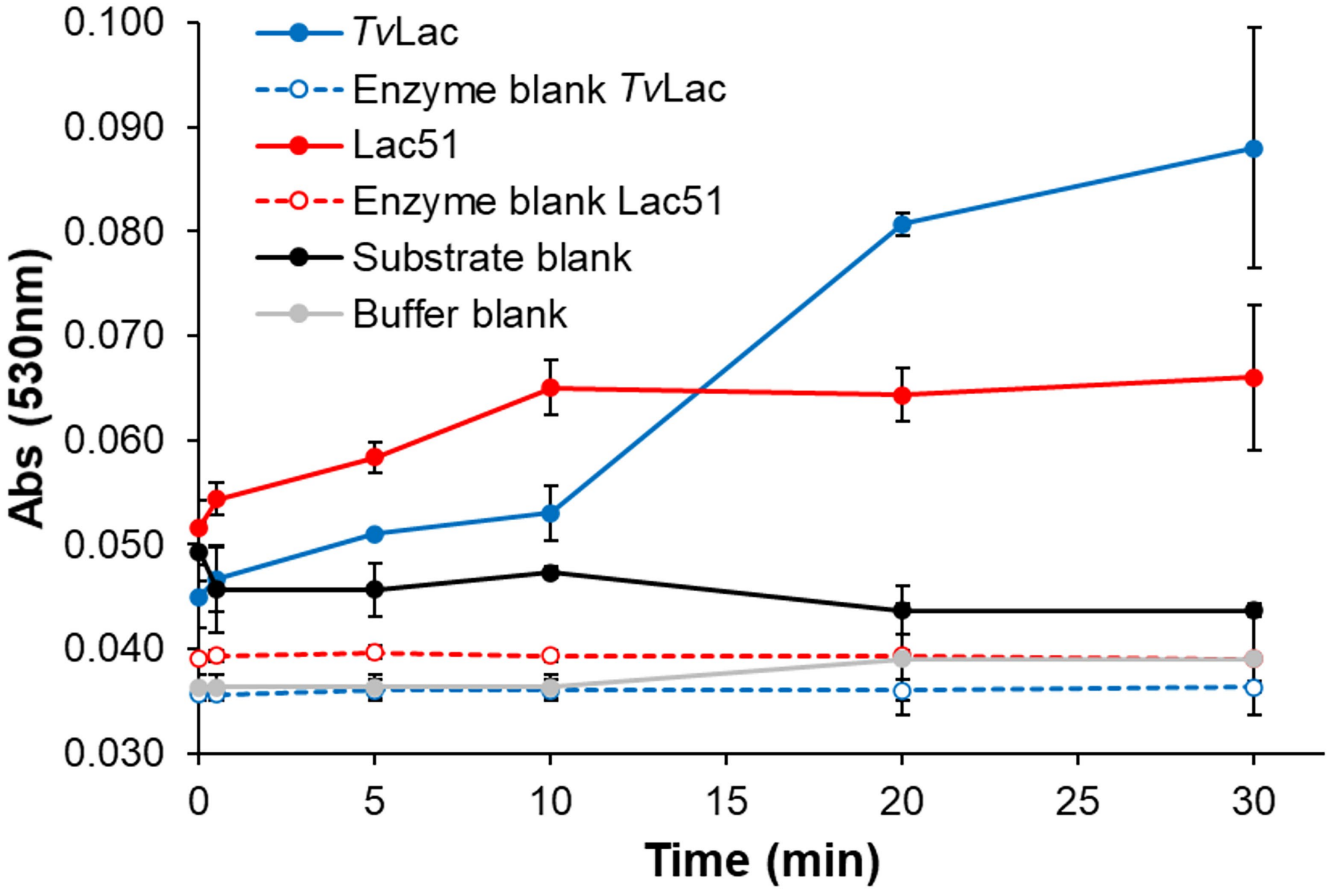
Laccase activity with syringaldazine. Syringaldazine (0.1 mM) was incubated with 0.4 μM Lac51 (red) or 0.2 μM *Tv*Lac (blue) in 50 mM Na-acetate buffer pH 5.0 at 37°C. The reaction was followed by detecting the formation of syringaldazine quinone at 530 nm. Substrate (black line) and enzyme blanks (dashed lines) were prepared by omitting enzymes and substrate, respectively; the buffer blank (gray line) lacked both enzyme and substrate. Error bars indicate SD of triplicates.

Expression of an active Lac51 protein in *N. benthamiana* was confirmed with the model substrate syringaldazine, using *Tv*Lac (*Trametes versicolor* laccase) as positive control. Lac51 was able to oxidize syringaldazine to its quinone form (indicated by an increase in absorbance at 530 nm), and in the first 10 min of the incubation, the activity of Lac51 was comparable with that of *Tv*Lac (Figure 3). However, while *Tv*Lac remained active up to 30 min (i.e., throughout the course of the reaction), the increase in the signal at 530 nm stopped after 10 min in reactions with Lac51, indicating inactivation of the enzyme.

**Figure 4:**
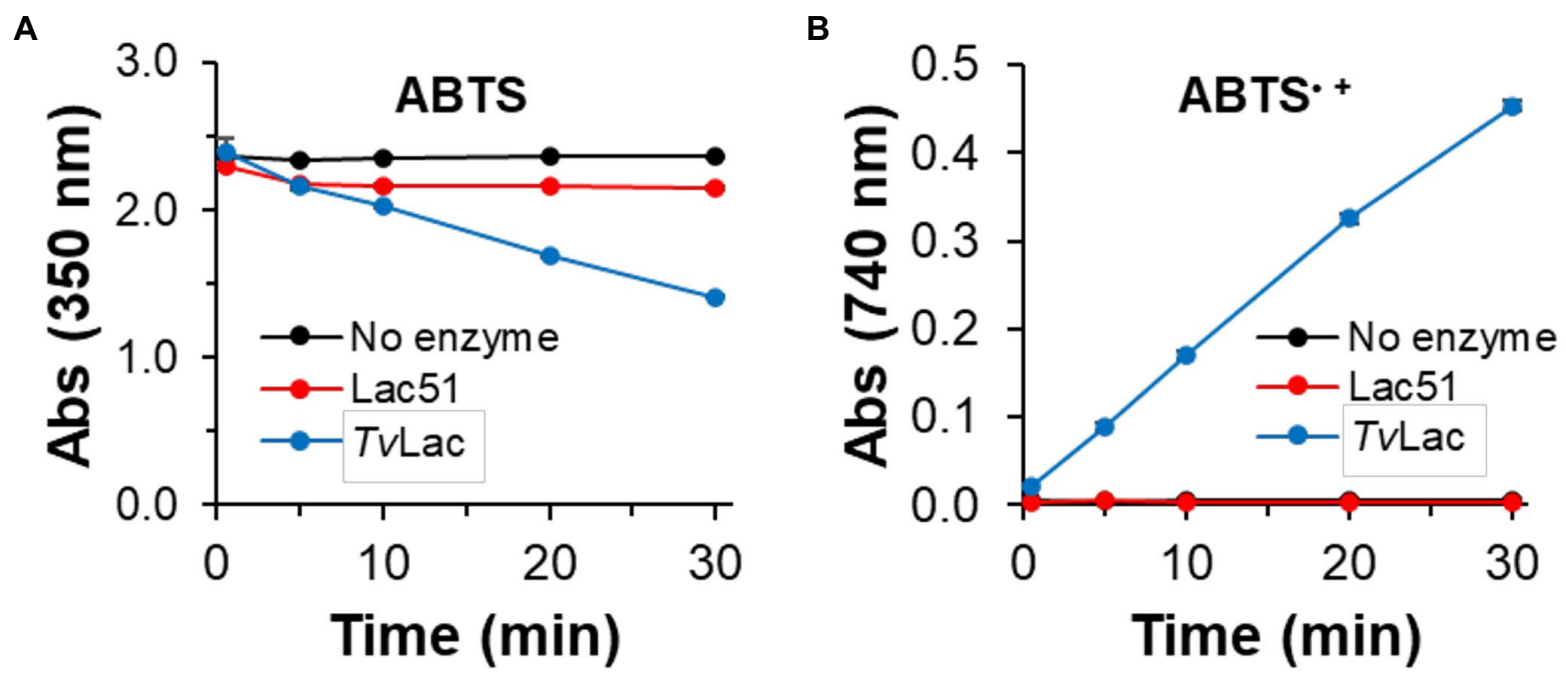
Laccase activity using ABTS. ABTS (0.1 mM) was incubated with 0.4 μM Lac51 (red) or 0.4 μM *Tv*Lac (blue) in 50 mM Na-acetate buffer pH 5.0 at 37°C. The reaction was followed by detecting **(A)** the consumption of ABTS at 350 nm and **(B)** the formation of ABTS^•+^ radical cations at 740 nm. Error bars indicate SD of triplicates.

Lac51 was also incubated with ABTS (Figure 4), which is regarded as one of the most sensitive substrates for detecting laccase activity (Li et al., 2008). Accordingly, the control laccase *Tv*Lac generated ABTS radicals at a constant rate (Figure 4B), which coincided with a decrease in the ABTS concentration (Figure 4A; Supplementary Figure S1). On the other hand, incubating ABTS with Lac51 led to only a small decrease in the concentration of the substrate within the initial 5 min, after which the ABTS concentration remained constant throughout the reaction (Figure 4A). In addition, Lac51 generated no detectable amounts of ABTS radicals (Figure 4B). It is noteworthy that the ligninolytic activity of two enzymes is best compared based on substrate consumption because laccases catalyze radical reactions and generate a broad range of products, some of which may be polymeric and, hence, insoluble in the reaction medium.

### Determination of pH and Temperature Optima of Lac51 Using Syringaldazine

**Figure 5:**
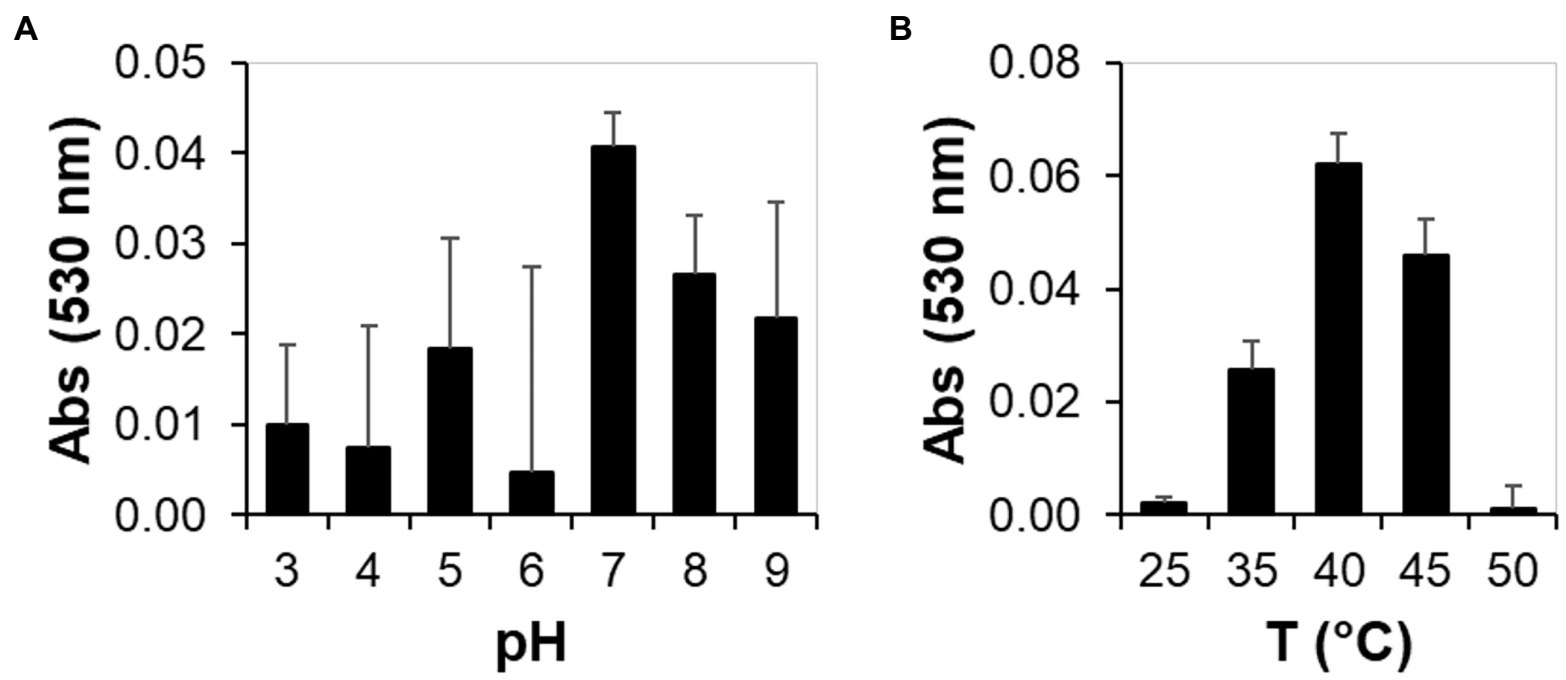
Biochemical activity of plant expressed Lac51 toward syringaldazine under different pH and temperature conditions. Syringaldazine assay was performed in different conditions containing 0.4 μM Cu^2+^-saturated Lac51, 50 mM Na-acetate, and 30 μM syringaldazine in a final volume of 0.1 ml with different pH **(A)** and temperature **(B)**. The absorbance was measured at 530 nm for over 30 min at 37°C.

As Lac51 showed measurable activity on syringaldazine, we used syringaldazine as a substrate and measured the rate of substrate consumption in the pH range of 3.0–9.0 and temperature range of 25–50°C. The optimal pH for the oxidation of syringaldazine was found to be 7.0, and the optimal temperature at pH 7.0 was found to be 40°C, as shown in Figure 5.

### Enzyme Activity on Lignin Monomers

**Figure 6:**
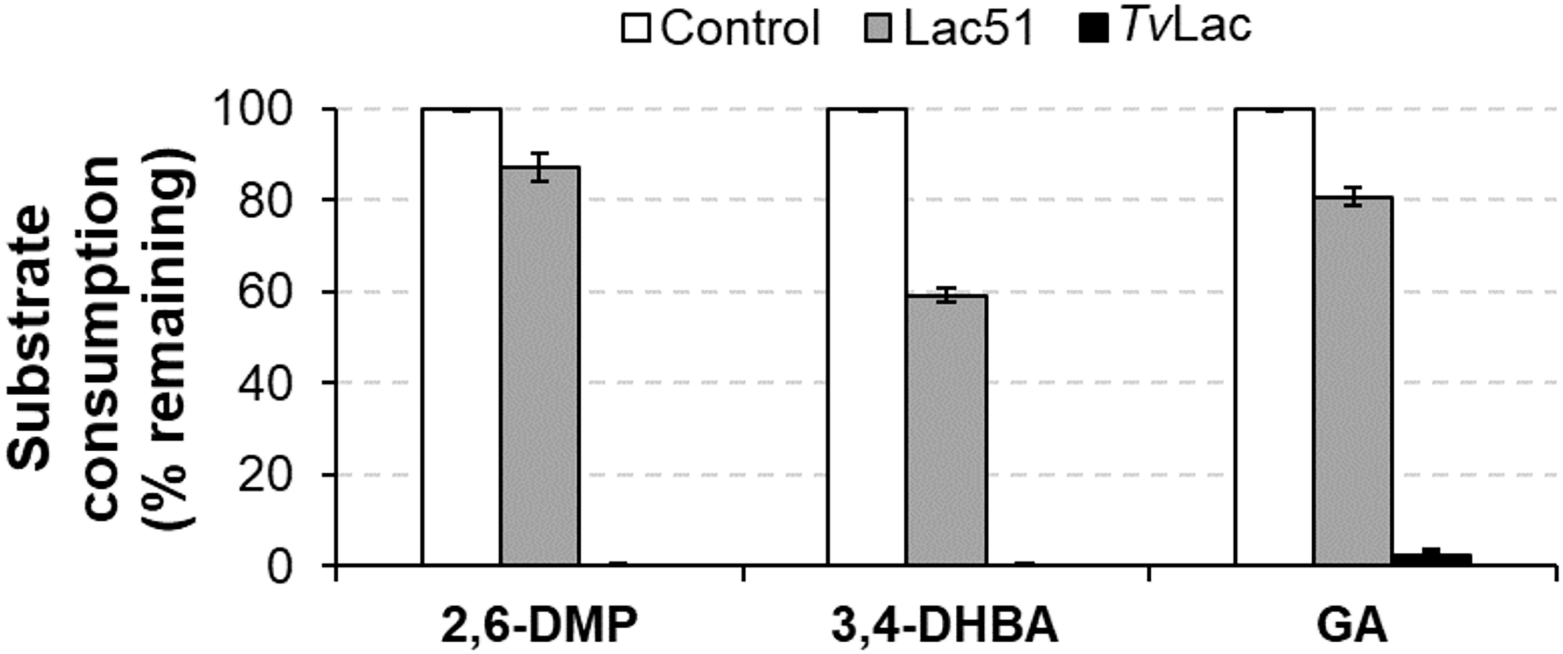
Activity of Lac51 and *Tv*Lac on 2,6-dimethoxyphenol (2,6-DMP), 3,4-dihydroxybenzaldehyde (3,4-DHBA), and gallic acid (GA). Lignin monomers (3 mM) were incubated with 2 μM Lac51 or *Tv*Lac in 10 mM BisTris-HCl buffer pH 6.5 at 37°C for 24 h. The reaction was followed by detecting the consumption of substrate at 315 nm for 2,6-DMP and GA and at 450 nm for 3,4-DHBA. The control reactions contained the substrates without enzyme. Reactions were run in triplicate; error bars represent SDs.

Lac51 and the positive control *Tv*Lac were tested for activity on seven lignin monomers. Spectrophotometric analysis revealed that Lac51 was active on three (2,6-DMP, 3,4-DHBA, and GA), while *Tv*Lac was active on five of these seven substrates [2,6-DMP, 3,4-DHBA, GA, vanillin (V), and guaiacol (G); Table 1]. After 24 h incubation, Lac51 oxidized 13% of 2,6-DMP, 41% of 3,4-DHBA and 19% of GA (Figure 6). *Tv*Lac, on the other hand, reacted more readily with these substrates, oxidizing completely 2,6-DMP and 3,4-DHBA, and leaving only 2% of GA behind in the reaction after 24 h. The action of both enzymes led to formation of insoluble residues in the reactions with 2,6-DMP and GA, presumably through polymerization of the lignin monomers.

**Table 1:** Activity profile of Lac51 and *Tv*Lac on lignin monomers.

Enzyme	2,6-DMP	3,4-DMBA	3,4-DHBA	4-MBA	GA	V	G
Lac 51	+	−	+	−	+	−	−
*Tv*Lac	+	−	+	−	+	+	+

Lignin monomers (3 mM) were incubated with Lac51 or *Tv*Lac in 10 mM BisTris-HCl buffer pH 6.5 at 37°C. The substrates are abbreviated as follows: 2,6-dimethoxyphenol (2,6-DMP); 3,4-dimethoxybenzalcohol (3,4-DMBA); 3,4-dihydroxybenzaldehyde (3,4-DHBA); 4-methoxybenzylalcohol (4-MBA); gallic acid (GA); vanillin (V); and guaiacol (G). A ‘+’ sign indicates modification of the substrate by the laccase. A “-” sign indicates negative modification of the substrate by the laccase.

Formation of soluble laccase reaction products was confirmed with LC-UV/MS^2^ analysis (Supplementary Figure S2; Table 2; Supplementary Table S1). The product profiles of the two enzymes, judged from both UV (Supplementary Figure S2) and MS profiles (Table 2; Supplementary Table S1) differed on all three substrates, which in part may be due to the differences in the extent of substrate conversion and/or polymerization. When subjecting the compounds detected by MS to MS^2^ fragmentation (Supplementary Table S1), we could identify signals corresponding to the oxidized dimer of 2,6-DMP (with *m/z* value 305.00 in Table 2). In the reaction of 2,6-DMP with *Tv*Lac (which led to complete conversion of the substrate, Figure 6), products accumulated mostly in the insoluble fraction, leaving virtually no low-molecular-weight components in solution, apart from small amounts of the oxidized dimer (Supplementary Figure S2A; Table 2). As insoluble polymer was also observed when treating 2,6-DMP with Lac51, albeit in a smaller amount than with *Tv*Lac, it is likely that the peaks observed in the reaction with Lac51 are the intermediate oligomeric products that are polymerized further to form the insoluble residue. Notably, the concentration of the oxidized dimer of 2,6-DMP (at 6.67 min in Supplementary Figure S2A) corresponded to the extent of substrate conversion by the two enzymes on 2,6-DMP (Figure 6).

**Table 2:** Reaction products of Lac51 and *Tv*Lac with lignin monomers identified with LC-UV/MS.

Substrate	Enzyme	Compounds identified		
		Retention time (min)	*m/z* (H^+^) Positive mode	Compound type
2,6-DMP	No enzyme	5.47	155.00	Monomer
		5.49, 6.67	305.00	Oxidized dimer
		5.49^a^, 6.67^a^, 7.75^a^	307.09	
	Lac51	5.47^a^	155.00	Monomer
		5.54^a^	374.00	
		5.49, 6.67	305.00	Oxidized dimer
		5.49^a^, 6.67^a^, 7.75^a^	307.09	
		−^b^	−^b^	Insoluble polymer
	*Tv*Lac	5.49, 6.27^a^	305.00	Oxidized dimer
		−^b^	−^b^	Insoluble polymer
3,4-DHBA	No enzyme	2.47	138.92	Monomer
	Lac51	2.47	138.92	Monomer
		4.71	257.09	
		5.04^a^	275.09	
		8.11^a^	451.09	
	*Tv*Lac	1.66^a^	468.09	
		2.18^a^	472.92	
		4.32^a^	466.09	
		4.71	257.09	
		5.04^a^	275.09	
		8.11^a^	451.09	
GA	No enzyme	0.82	170.92	Monomer
	*Tv*Lac	−^b^	−^b^	Insoluble polymer
	Lac51	−^b^	−^b^	Insoluble polymer

Lignin monomers 2,6-DMP, 3,4-DHBA or GA (3 mM) were incubated with Lac51 or *Tv*Lac in 10 mM BisTris-HCl buffer pH 6.5 at 37°C for 24 h. For the UV chromatograms, see Supplementary Figure S2.

a Products with no UV absorbance in Supplementary Figure S2.

b Insoluble polymeric products were not detected with LC–MS as they were filtered out of solution before loading the sample on the column.

## Discussion

In the current study, we assessed transient expression of a bacterial laccase, Lac51, originating from *Pseudomonas putida* and identified in the giant panda’s gut, in the non-food plant *N. benthamiana* using a vacuum infiltration-based system, providing a low-cost alternative to bacterial and eukaryotic expression hosts. Plants or plant cells/tissues have been used to express valuable recombinant proteins, including vaccines, enzymes, and biopharmaceuticals in molecular farming and have been established as an economically viable (and scalable) alternative to mainstream production systems (Clarke et al., 2013; Bock, 2015; Buyel et al., 2017). In general, nuclear or transient expression is preferred for expression of eukaryotic proteins, mostly when post-translational modification (e.g., glycosylation) of the target protein is a requirement for protein functionality or stability, whereas plastid expression is often the more common choice for expression of bacterial proteins (Xu et al., 2012). Plants have also been exploited for expression of different plant cell wall-active enzymes, including laccases derived from plants and fungi (Hood et al., 2003; Wang et al., 2004; Sonoki et al., 2005), but not laccases of bacterial origin (Preethi et al., 2020). Plant genetic engineering technologies have expanded the diversity of well-established plant-based bioproduction platforms (Paul and Ma, 2011; Xu et al., 2012) and also expressing systems, while stable transgenic lines display high yield of the final product and transient expressing plants exhibit fast processes (Gecchele et al., 2015).

In this work, the recombinant His_6_-tagged Lac51 was successfully cloned and transiently expressed in *N. benthamiana*, and the plant-derived Lac51 was shown to be produced in a catalytically active form. Notably, protein instability affected Lac51 accumulation over time, showing the impact of harvest time on the protein yield. Overexpression of plant cell wall-degrading enzymes especially in plant chloroplasts, which is often used for bacterial enzymes, can be a severe physiological burden for the host plant and limit plant growth (Oey et al., 2009). Apart from minor alterations in the leaf tissue (as compared to the RFP control), this was not the case during transient expression of Lac51 (Figure 1). The present results show the capability and potential of plant produced enzymes, that, however, still needs further research.

The catalytic activity of the plant produced Lac51 was compared to that of a commercially available fungal laccase *Tv*Lac from *Trametes versicolor* (this study) and to literature data for a recombinant Lac51 expressed in *Escherichia coli* [an earlier study by Fang et al. (2012)]. In general, the plant-expressed Lac51 was found to have similar substrate specificity as well as similar pH (7–8) and temperature (40–45°C) optima on syringaldazine compared to the *E. coli*-expressed variant (Fang et al., 2012). Differences in apparent operational stability may be attributed to differences in reaction conditions, the reaction time in particular [5 min vs. 30 min in Fang et al. (2012) and this study, respectively]. When comparing the (plant expressed) bacterial Lac51 to the fungal *Tv*Lac (this study), there were clear differences in substrate specificity and operational stability between the two enzymes under the tested conditions. In general, *Tv*Lac acted more readily with the tested substrates (Figures 4, 6) and retained its activity over a longer time period (Figure 3). The differences in operational stability (Figure 3) and possible mode of action (see Figure 4) indicate that laccases need to be selected carefully for targeted applications.

In summary, we transiently expressed a bacterial laccase originating from a giant panda gut microbe, denoted as Lac51, which is the first functional recombinant laccase produced in plants. The plant-expressed Lac51 showed enzymatic properties and lignin degradation potential similar to the *E. coli*-expressed variant reported earlier (Fang et al., 2012). Thus, our study demonstrates successful production of a catalytically active bacterial laccase and further shows the potential of green and non-food plants e.g., *N. benthamiana* as an economical enzyme production platform for the future production of bacterial enzymes.

## Data Availability Statement

The original contributions presented in the study are included in the article/Supplementary Material, further inquiries can be directed to the corresponding author.

## Author Contributions

AE, AV, HE, VE, and JC conceived and designed the study. LP, YW, and FQ carried out vector design, sequencing and participated in agroinfiltration. YW and AE performed protein purification and analysis, J-KJ analyzed enzyme properties. AE, YW, AV, HS, and JC drafted the manuscript. All authors contributed to the article and approved the submitted version.

## Funding

This work was supported by the NIBIO core funding “ArcGene” project 11145 and the Research Council of Norway, through grants 243974 (Bioboost) and 270038 (NorBioLab).

## Conflict of Interest

The authors declare that the research was conducted in the absence of any commercial or financial relationships that could be construed as a potential conflict of interest.

## Publisher’s Note

All claims expressed in this article are solely those of the authors and do not necessarily represent those of their affiliated organizations, or those of the publisher, the editors and the reviewers. Any product that may be evaluated in this article, or claim that may be made by its manufacturer, is not guaranteed or endorsed by the publisher.
