# Supplementary material for: Successful Production and Ligninolytic Activity of a Bacterial Laccase, Lac51, Made in Nicotiana benthamiana via Transient Expression
Source: Front Plant Sci. 2022 May 13;13:912293. doi: 10.3389/fpls.2022.912293 (PMC9141054; doi:10.3389/fpls.2022.912293)
Supplement: Supplementary file 1 [file Data_Sheet_1.docx]

## Supplemental materials


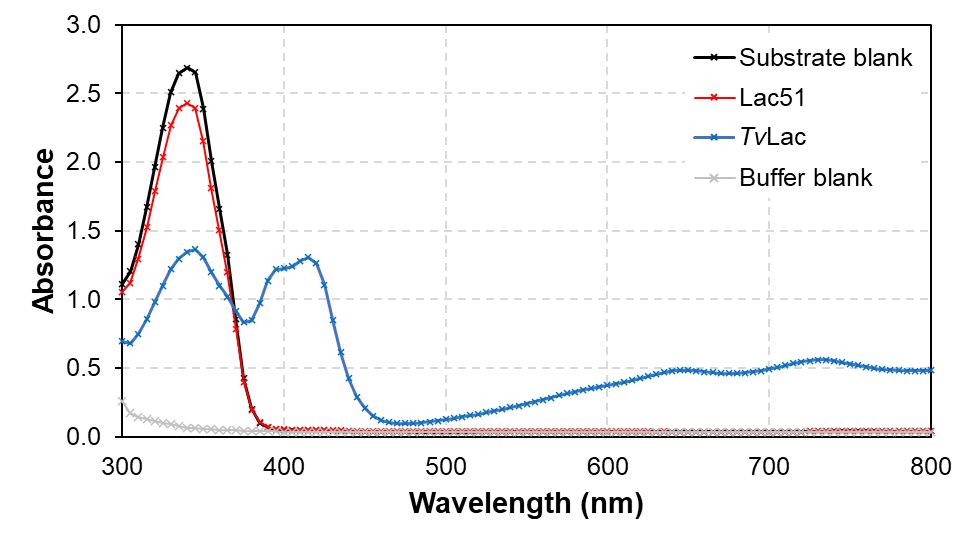


## Fig. S1 Spectral scans of reactions with ABTS after 31 min enzyme treatment.


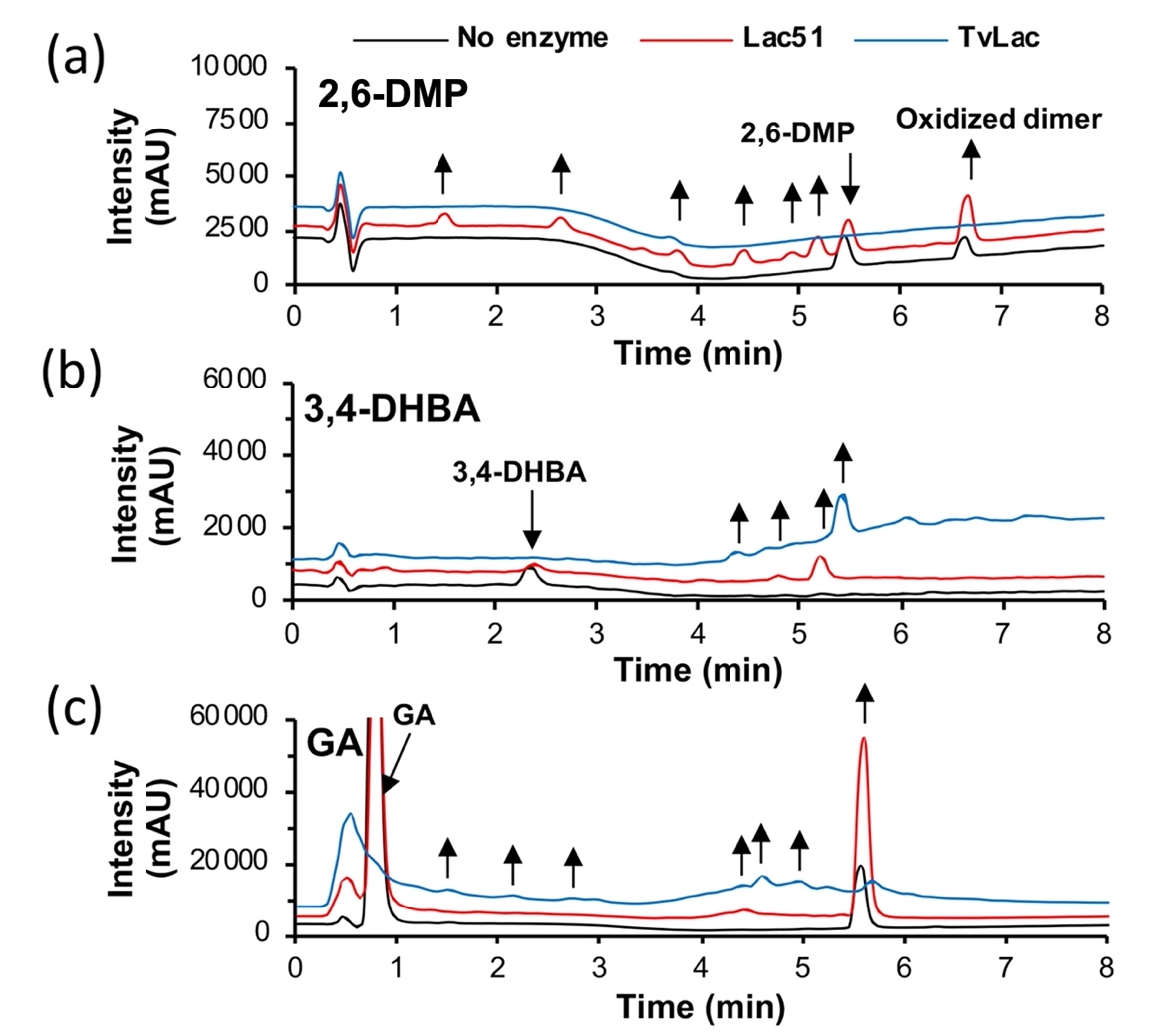


**Fig. S2** Product profiles of Lac51 and *Tv*Lac incubated with lignin monomers. Lignin monomers (a) 2,6-DMP, (b) 3,4-DHBA or (c) GA (3 mM) were incubated with Lac51 (red lines), 2 µM *Tv*Lac (blue lines) or no enzyme (black lines) in 10 mM BisTris-HCl buffer pH 6.5 at 37 °C for 24 h. UV chromatograms were recorded at 315 nm for 2,6-DMP (a) and GA (c) and at 450 nm for 3,4-DHBA (b). Substrates are marked with downward arrows; accumulation of products is indicated with upward arrows.

## Table S1 MS^2^-fragmentation of the laccase reaction products listed in Table 2. The products that were identified based on MS^2^ data are indicated. For the fragmentation data, the mass loss is given in brackets.

| **Substrate** | **Enzyme products** | |  |  |
| --- | --- | --- | --- | --- |
| **Name** | **Retention time**  **(min)** | **MS**  ***m/z* (H^+^)**  **Positive mode** | **MS^2^**  ***m/z* (H^+^)**  **Positive mode** | **Compound** |
| **2,6-DMP** | 5.54 | 374.00 | 351.00 (-23)  333.00 (-41) |  |
|  | 5.49, 6.27, 6.67 | 305.00 | 287.00 (-18)  277.00 (-28)  262.00 (-43)  245.00 (-60)  213.00 (-92)  185.00 (-120)  157.00 (-148)  129.00 (-176) | Dimer  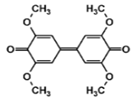 |
|  | 6.67, 7.75 | 307.09 | 275.00 (-32)  243.00 (-64)  153.00 (-154) |  |
| **3,4-DHBA** | 1.66 | 468.09 | 432.09 (-36)  192.00 (-276)  174.00 (-294) |  |
|  | 2.18 | 472.92 | 436.09 (-37)  410.09 (-63)  392.09 (-81)  374.09 (-99)  350.00 (-123)  192.00 (-244) |  |
|  | 4.32 | 466.09 | 438.84 (-28)  430.09 (-36)  367.92 (-99)  192.00 (-274) |  |
|  | 4.71 | 257.09 | 229.00 (-28)  215.92 (-41)  211.00 (-46)  201.00 (-56) |  |
|  | 5.04 | 275.09 | 247.00 (-28)  229.00 (-46) |  |
|  | 8.11 | 451.09 | 395.00 (-56)  377.00 (-74)  331.09 (-120) |  |
